# Personal Observations of the Development of Dentistry and Dental Legislation in Michigan

**Published:** 1896-11

**Authors:** G. E. Corbin


### Personal Observations of the Developement of Dentistry and Dental Legislation in Michigan.

BY G. E. CORBIN, M. D., D. D. S.

History, with names and dates omitted, would be tame and valueless.

As this article is to be a brief history of personal observations, names and dates are a necessity, but in their use I hereby disclaim all intent or desire to deal in any terms that may, in any way, be construed or misconstrued as personalities. While pursuing the study of medecine at the University of Michigan in the autumn of 1853, I found myself at the same boarding-house with one Seth A. Gerry. In his side coat pocket carried a small roll of instruments, consisting of a few scalers, excavators, hand-pluggers and a turnkey. With these, he said, he had easily and rapidly earned money enough to pay the expenses of a medical education—to which he aspired.

He asserted, that with ten dollars' worth of instruments, and the information that he could impart in one week's time—and that even without encroaching upon lecture-hours—I could go to small towns, among strangers, and earn from $5 to $15 per day without any sort of trouble. Though I had already put in one full year at hard study, including one course of lectures, I expressed the opinion that I was too deficient in the knowledge of anatomy, physiology. pathology, etc., to engage in such a practice. He said that, with a hammer, I could crack open and inspect a few teeth, and in twenty minutes learn all the anatomy necessary. His kindness of heart can be better understood when 'tis known that all he proposed to charge for the week's instruction was fifty dollars. He found the medical course too laborious, dropped out, and resumed the practice of the profession he acquired so easily.

A little later I made the acquaintance of Chas. H. Sackrider, a very bright and genial student, who, after graduation, practiced the profession of medicine and surgery very successfully for many years at Mason, in Ingham County. Sackrider had previously conducted a lucrative practice of dentistry in Canada, and had the money to show for it.

Think of the change in Canadian laws since that date! Now no one is permitted to practice in Ontario until he has passed a satisfactory examination before its Board of Examiners. More than that, no one is permitted to take the examination unless he be a graduate of the Royal College of Dental Surgeons of Ontario. However well qualified, foreigners, evidently, are not wanted there. A large number of Canadians, however, both graduates and non-graduates, find it convenient to locate in Michigan, where, both from experience and observation, they have learned that they will not be likely to be seriously annoyed either by examinations or dental laws.

In the "early fifties" one A. J. Wiggins located in Chelsea for the practice of medicine, and stocked his office liberally for individual prescribing. A few years afterward he exchanged the remnants of his drugs for the unused and rusty dental instruments owned by Dr. Sackrider. The possession of these instruments caused Dr. Wiggins to travel about the country, for a time, extracting and filling teeth, and then he called himself a dentist. The knowledge thus acquired caused him to engage quite extensively in the occupation of manufacturing dentists at $50 per head. The time required for this transformation varied from one to three weeks, according to the adaptability of the subject, or more accurately, the doctor's ability and success in making the collection. Many so-called dentists were thus started out in the business.

An occasional graduate from the dental colleges at Cincinnati, Baltimore and Philadelphia, and able and conscientious practitioners from other sources, located in the State. There was no law to prevent and no law to regulate. Then, as now, there were good, bad and indifferent—and worse than either.

With no dental school in the State, it was quite the custom for representative dentists to receive in their offices conscientious young men for instruction, who were obligated to remain for a specific time, usually two years. In these various ways the the ranks of the profession were augmented.

In 1853 the idea of mounting teeth upon a rubber plate was first successfully accomplished. The process was easily learned. It was described in a printed circular which accompanied each vulcanizer sold. This mode of advertising vulcanizers was a success. Prior to this date each plate of teeth mounted on a gold base had cost $100, or more. The persons who could construct really meritorious gold plates were not numerous. Those who were proficient in the process of manufacturing continuous gum-work were less numerous.

In those days the replacing of lost teeth involved much expense. More than this, the mutilation in the loss of natural teeth was mortifying, humiliating, so that dentists were enjoined to the strictest secrecy.

Alas! how changed! Rubber plates of all possible grades of merit could be furnished at less than one-half the price of gold ones and still return better pay for the labor expended. This state of affairs speedily flooded the entire country with young, aspiring, bungling mechanics, who resorted to every means possible to induce human beings to part with good, natural teeth, that they (the mechanics) might get pay for inserting bungling substitutes.

In the profession of dentistry, as in that of every other respectable avenue of life, are to be found conscientious and capable men. At the date now under discussion these men stood aghast! Their sympathies and sorrows were aroused by a knowledge of the impositions practiced upon an unsuspecting community. Their professional pride was shocked at having such unprofessional conduct charged up to an honorable and very necessary profession. What was to be done? What could be done? It was wisely decided that by united action something could be accomplished in the direction of educating the community to a better understanding of the facts in the case. To this end, and for the purpose of mutual benefit in the exchange of ideas, the Michigan State Dental Association was organized; and it has rendered much valuable service. Largely, though not entirely through its influence, the dental department of the University of Michigan was established.

No government, founded on the principle of universal suffrage, can long survive, unless the votes be intelligently cast. "A government of the people, for the people, by the people," necessarily implies and carries with it a system of popular, public education.

There is scarcely a dissenting voice upon the question of compulsory education in the early years of childhood. Upon the question of taxation of all the property in the State to aid in the education of professional men, strong arguments may be advanced both *pro* and *con*, but the State of Michigan long since decided that question for herself, and in the affirmative. She has provided the best of facilities for the education of her teachers, lawyers, doctors, pharmacists, dentists and others. Michigan yearly expends a large amount of money that her citizens, one and all, from one end of the State to the other may avail themselves of the best of talent in the directions mentioned.

That these citizens may not be imposed upon by incompetent pretenders, legislation to this end has been found necessary. Teachers, lawyers, pharmacists and others must all pass required examinations before they can legally practice their respective callings. The first law passed "to regulate the practice of dentistry in the State of Michigan" was Act No. 140, Laws of 1883. This law provided for a Board of Examiners, but it admitted to registration all who were then in practice in this State, on filing an affidavit to that effect, and the payment of a registration fee of 25 cents. It also legalized the practice of all persons holding "diplomas from the Faculty of any reputable dental college, duly incorporated under the laws of this or some other State of the United States."

Under the wording of this law the possession of a diploma from a college of the very shortest term and lowest grade, legalized the practice of the holder regardless even of registration.

In those days there was a regularly-chartered dental college at Delevan, Wisconsin, conducted by one Morrison, that issued diplomas (to start with) at $12.50 each, express charges prepaid. Later, as business flourished, the price was raised to $25.00 each, showing a great financial advantage to those who applied early. From this college, that sold diplomas and delivered them by express, without any attendance whatever, up to those requiring attendance of three full years of nine full months each, upon a most complete and thorough curriculum, there were colleges of almost every conceivable intermediate grade, whose diplomas, under the first dental Act, placed all alike upon an equality before the public.

Another, and a serious defect in the original act, was in the language that permitted the Board to collect the prescribed fee for examinations from successful applicants only. The incompetent ones required the most time and paid nothing, so that for several years the receipts were not sufficient to meet the unavoidable expenses, and much gratuitous work was necessarily done by the Board. Defective as this first act was, much valuable service was rendered under it in the direction of organization, examination and registration. Especially to cure these two last-named serious defects the law was amended in 1891 by the alteration of

Sections 1, 7 and 9, and the addition of two new sections. As thus amended the law very properly established and defined a standard of attainment to be required by the Board.

The laws of thirteen States of the Union recognize no diplomas whatever, but exact examinations of all applicants. Several of the States, like Ontario, exclude all from examinations even, unless they be graduates. Not so with Michigan; she is much more generous. All applicants are admitted to examination, and all who are qualified are sure to be licensed and registered; but the unqualified, unlicensed, illegal practitioners in the State are not sure to be prosecuted. This fact has become too well known. None but dentists are at all likely to know; very few but dentists have any knowledge of the process by which they *might* know who are the qualified, registered, legal practitioners, and who are not. For this reason none but dentists can reasonably be expected to enter complaints and enforce prosecutions. Nowhere in the law is there any other provision for its enforcement.

In the enactment of this law the people of the State of Michigan very evidently expected this much of the dental profession. 'Tis asking none too much. Unfortunately, however, what is everybody's business is generally conceded to be nobody's business. Each waits, and expects of his neighbor what he is not quite willing to do himself. Nothstanding this general feeling much has been done under this law. I have no way of ascertaining the total number of prosecutions had under its provisions, but it may be profitable to briefly review a few of them: Wm. F. Alton was arrested at Imlay City, taken to Lapeer, and on January 7, 1890, tried, convicted and fined $25 for the illegal practice of dentistry. He paid the fine, returned to Imlay City and to his practice. For this reason, on March 27, 1891, he was again tried and convicted, and a second fine of $30 was inflicted. This closed his practice in the county of Lapeer, but whether he resumed practice elsewhere in the State I am not advised.

In the month of February, 1894, in the city of Grand Rapids, T. S. Hudson was arraigned on the charge of illegally practicing dentistry. An adjournment was procured, during which he secured a "temporary license" from a member of the State Board of Examiners in Dentistry, which, of course, quashed the suit. This was certainly very unfortunate—unfortunate for law and justice; unfortunate for those who were trying to enforce a wise and just law; unfortunate for the "people of the State of Michigan," who, through their representatives, enacted the law; unfortunate for T. S. Hudson himself; unfortunate for any man to be sustained or encouraged in evading, violating or defying any just law of his country or the State; unfortunate for the Board of Examiners in Dentistry; unfortunate for the member issuing the temporary license; unfortunate for all the persons who conspired in the misrepresentation and deception practiced upon him to procure this "temporary license"; unfortunate for a man who is distinguished for kindness of heart and unswerving fidelity to duty.

I have been informed, on the best authority, that when E. B. Crandell, of this city (Grand Rapids), was put on trial for illegally practicing dentistry, he was immediately dismissed or discharged by the judge at the request of his attorney, simply and solely for the reason that the very first question propounded to the complaining witness elicited the fact that he (the witness) was not personally acquainted with said Crandell! Reflect on that fact for a moment, please! Can you think of anything more preposterous? Where is there another judge on earth who would be willing to put himself on record for such a ruling? Think of a ruling, a precedent that will prevent you from entering complaint against a burglar; against a man for committing theft, arson or any other crime or misdemeanor, unless the offender happens to be a personal acquaintance! How ridiculously absurd!

I have consulted a competent lawyer on that subject. He informs me that statutory law requires a justice to issue a warrant, on complaint, whenever accompanied by sufficient evidence from whatever source, by whomsoever produced, to convince him that there is a reasonable probability of the guilt of the party complained of; that is, enough to start proceedings to determine the fact by a jury on presentation of the evidence.

I here quote from "Tiffany's Criminal Law," fourth edition (1389), page 36. He there says:

"In general, every man is, of common right, entitled to prefer an accusation against a party whom he suspects to be guilty of a criminal offense.

"Those only are disqualfied from becoming complainants who are incompetent to become witnesses."

The cases Tiffany cites as incompetent to become witnesses are cases of insanity, drunkenness, extreme age and infirmity, extreme youth, etc., and cases where husband and wife can not "complain against each other," none of which apply to the case under discussion.

Beyond all question the complaint was legally and properly made, and it was an unwarrantable proceeding to discharge the offender without trial.

Tiffany still further says: "All persons legally entitled to prefer an accusation against a party suspected of crime are bound to exert the power with which they are invested."

That, gentlemen, reaches us all—every one of us. We are not only permitted, but by the law commanded, to aid in the enforcement of all just laws by making proper complaints of all known infractions of them. When we fail to do it we shirk the duty imposed on all good, law-abiding citizens. We must all do that much, and if the ends of justice are thwarted in the machinery of the courts, it can not be charged up to our fault or neglect. Justice is certainly not always attained in courts at law. The definition of a court-house may well be given as a place where—*in*-justice is dispensed.

This case occurred more than two years ago, and the culprit is still practicing in this city in open violation of the law! How many of you, gentlemen, are conscience-clear under the express commands of the law in this case? Let us suppose a case. Let us suppose that this was a civil suit brought by any one member of the Grand Rapids Dental Society for the collection of a claim of ten dollars, ruled out of court because of some technical error in the wording of the summons, is there anybody here who doubts that the defendant would have been promptly brought before the court the next morning on an amended complaint?

In December, 1894, on complaint of the writer hereof, one

M. D. Halsey was put on trial at St. Johns, Michigan, for practicing dentistry illegally. Very briefly stated, the facts are these: He advertised, as his exclusive business, that of extracting teeth, and proceeded to do so. The law makes the practice of dentistry illegal without first proving qualification and procuring registration on such proof.

Halsey had nót complied with the law in this matter, and so acknowledged. He claimed the right to engage exclusively in the business of extracting teeth, as he had been doing for the last preceding four years, because, for twenty years prior to that date he was a practicing physician and surgeon. This claim was based on a clause in the law which reads as follows: "Nothing in this act shall be construed so as to interfere with physicians and surgeons in their practice as such," that is, in their practice as *physicians* and *surgeons*. That language is certainly clear. The only question in the case, was, whether he was engaged in the practice of general surgery or of dentistry?

Three of the leading physicians, of St. Johns, constantly engaged in the general prctice of medicine and surgery were called by the defense, and, very properly, testified that they frequently extracted teeth, and thought they had the right to do so. On cross-examination, being both intelligent and honest, each testified that a man engaged exclusively in the business of extracting teeth would not be practicing general surgery, but that his business would, necessarily, have to be classed as "dental surgery."

Webster's definitions, which were read to the jury, make "dental surgery" synonymous with the single word *dentistry*, which can not be legally practiced in Michigan without first procuring registration, which Halsey acknowledged he had not done. This was the whole case, and so plain, so clear, so distinct, that a bright ten-years old school boy, if awake, could not fail to correctly understand it. The above facts were submitted to a jury, at least one of whom sat and nodded, blinked, and slept through most of the trial! This jury returned a verdict of "no cause for action!"

In a trial at law, either civil or criminal, it is discouraging, exasperating, to obtain a verdict which is entirely at variance with law, evidence and justice. In this case, however, there is another side to be observed. The first jury disagreed, making two trials necessary, each of which cost the defendant twenty-five or thirty dollars and the people of the county of Clinton a small fractional part of a cent each. He could have been arrested again and again for the very next, and each subsequent infraction of the law, whatever the decision of any jury in the case. The one experience in Clinton county, however, had been rendered unprofitable and dangerous to repeat; accordingly, he packed his grip and left for parts unknown.

In March last, Archibald Cunningham was put on trial, in Detroit, for violating the law to regulate the practice of dentistry in Michigan. In reporting this case the Detroit *Free Press* stated that Cunningham's lawyer, one Glidden, "claimed that the law under which the complaint was made is unconstitutional, as it makes a distinction between a citizen of this and other States." One thing is certain, and that is, that the constitutionality of the law is not on trial before *any* jury. He might unjustly, improperly influence the jury by dragging in that question, but it has no business before the jury, whatever. That the dental law is unconstitutional, for any reason, whatever, I do not believe; that it is unconstitutional for the reason assigned by attorney Glidden, I most emphatically deny. If the law means anything it means just simply and exactly what it says, no more, no less. It is written in plain, simple, easy English words that we all understand; in words that we can comprehend just as well as can any lawyer. Study the law carefully, each one of you for yourself, and you can not find one sentence, or a clause of a sentence, to justify the assertion of attorney Glidden. During my whole seven years services on the Board of Examiners there was not a single interpretation, act or decision by the board to justify attorney Glidden's assertion.

The law does discriminate against the graduates of dental schools of foreign countries. The law, very justly, does discriminate between long term and short term, high grade and low grade dental colleges. It fixes a standard of comparison. It fixes a degree of qualification necessary to permit the party to legally practice dentistry in this State. The applicant has either one of two ways at his disposal by which to prove his qualification. Any person, from any State in the Union, presenting himself with a diploma from any dental college in the United States of the required and stipulated standing, is entitled to registration without examination. All other applicants, from whatever State or nation, are required to prove qualification by submitting themselves to an examination before the board. That is all. The matter of nationality, State or citizenship, does not enter into the question under the law. The most careful scrutiny of the entire law will not reveal any thing of the sort. More than all this we have high legal authority for the assertion that the law is constitutional.

In December, 1892, while the board was in session, at Detroit, Dr. Charles A. Emerson and Dr. Henry L. Weak, presented themselves before the board and demanded registration on their diplomas which was refused, for the reason that the colleges from which they graduated did not have "courses of instruction and practice fully equal or equivalent to that of the college of Dental Surgery of the University of Michigan," as the law requires. By agreement, an hour was named at which time they returned, accompaniod by their attorney, one Peter E. Parke, who argued the case at some length, citing various reasons why he considered the law weak, and even unconstitutional.

His argument and citations were taken verbatim by a stenographer employed by the Board for the occasion, and referred to the Attorney-General of the State for his opinion, which I will now read :

"STATE OF MICHIGAN, ATTORNEY GENERAL'S OFFICE,
"LANSING, February 22, 1893.

"*A. T. Metcalf, Esq., Secretary of the State Board of Examiners*
"*in Dentistry, Battle Creek, Michigan :*

"DEAR SIR: Your favor requesting my opinion as to the validity of Act No. 98, of the Public Acts of 1891, which is an Amendment to Act No. 140 of the Laws of 1883, entitled 'An Act to Regulate the Practice of Dentistry in the State of Michigan,' and as to the right of the said Board of Examiners to exercise their discretion and sound judgment under said law in determining what colleges have a course of instruction and practice fully equal or equivalent to that of the College of Dental Surgery in the University of Michigan, is received and considered.

"*I am very clearly of the opinion that the law is constitutional.*

"The State has a perfect right to prescribe the qualifications of persons who practice dentistry within its borders. I think no one would question the right of the State to require every applicant who was not a graduate of the College of Dental Surgery of Michigan to submit to an examination. But we have been more liberal than this, and have admitted graduates of certain other colleges without examination.

"Such a law is not in violation of the Fourteenth Amendment of the Constitution of the United States, which provides that no State shall deny to any persons within its jurisdiction the equal protection of the law. (See Bradwell vs. State, 16 Wallace, page 130; State vs. State Medical Board, 32 Minnesota, page 324.)

"In as much as the State has a right to prescribe the qualifications of the dental surgeons who desire to practice dentistry within its borders, the power of determining such qualifications must be placed with some Board or officer, and the Legislature has very wisely left this matter with a Board especially qualified in such matters, and when said Board has acted fairly and has justly exercised the discretionary power reposed in it, no one can legally complain. (State vs. Gregory, 83 Mo., 123; State vs. State Medical Board, 32 Minn., 324; Ambler vs. Auditor-General, 36 Mich., 271; "Mechem's Public Offices and Officers," Sec. 945; Board of Supervisors vs. Auditor-General, 27 Mich., 165.)

"The law leaves it with the Board to determine what colleges have a course of instruction and practice fully equal or equivalent to the College of Dental Surgery of the University of Michigan, and they having determined what colleges do have a course of instruction and practice fully equal or equivalent to that of the College of Dental Surgery of the University, and it not appearing in any way that they have abused their discretion in this regard, I nor any other person should presume to substitute our opinion as to what colleges are, or are not, entitled to this distinction.

"I give it as my opinion, therefore, that the Board has a right, when acting fairly within the discretionary power vested in it by law, to determine what colleges have a course of instruction and practice fully equal or equivalent to that of the College of Dental Surgery of the University of Michigan, and they can refuse to register applicants in accordance with such determination. "Respectfully,

(Signed) A. S. ELLIS, *Attorney-General.*"

The intent of the dental law is good. Its terms are liberal. All who are qualified can easily prove the fact, procure registration, and thereby legalization. Beyond all question its language, terms, conditions, requirements are all within the prescribed constitutional bounds, both State and National. Its rigid enforcement would accomplish the object sought in its enactment.

The prosecuting-attorney of each county, by virtue of his oath of office, is directly pledged to attend to all cases brought under this law. Some are both willing and competent, and some have proved themselves very loth to discharge their duties in the matter, a reason *sometimes* assigned being that their offices are salaried and that they get just as much for doing nothing as for doing their duty.

The law designates no person whose duty it is to make complaints or to collect and produce evidence. The people in general, whom the law was enacted to protect, can not make the complaints, for they have no knowledge as to whom the offenders are. The representative dentists of the State are the persons on whom this duty certainly devolves, and they have, inexcusably, shirked this duty. Why? Echo answers, "Why? If it be from extreme individual modesty or bashfulness the obstacle may, perhaps, be overcome by united action as represented by this Association. Certainly, for many long years it has done its duty and has done it cheerfully, and nobly, and well. I believe it can be depended upon in this emergency. What has been "everybody's business has been nobody's," and during the management of Nobody the illegal practitioners, as reported to the Board of Examiners, have increased year by year from a very few up to 205, the present number on the list. If, instead of reporting these to the Board, the dentists making the reports had reported to the proper officers, and caused prosecutions, as was their duty, the present number would have been reduced by about 200. The registry list now contains 825 names of legal practitioners, but there is no provision in the law for correcting this list. Of course many have died, and many have removed from the State since this list was commenced thirteen years ago. Probably the total number now in practice will not exceed 700. Not less than one in five of all now in practice throughout the State are doing so illegally. Such of these as are qualified should be registered, and the others should be compelled to suspend practice.

There are very few illegally in the practice of pharmacy. The pharmacy law expressly stipulates that the Board shall investigate all complaints and prosecute all offenders.

What is compulsory on the Board of Examiners in pharmacy can be voluntarily done by this Association in relation to violators of the dental law. It has been suggested that this Association employ an attorney to look after these matters. In what respect an attorney, drawing a salary from this Association, would be better than one drawing a salary from the county, is not clear. 'Tis true that many "snide" dentists are paid for worthless work, but conscientious, representative dentists receive pay only for their successes—never charge for their failures. I would suggest that an attorney be employed on the same basis. Pay him a reasonable fee for each success—for each conviction—and nothing unless conviction be secured. On such a basis he will study his cases well, and will make a success of it.

I would suggest that this city, and the city of Detroit, be first canvassed and cleansed. I would employ an attorney on the basis suggested above for *each* city, and set them at work at home, which will save all traveling expenses. By the time each city shall have been purged, a general exodus will purify most of the State.

Dr. A. W. Diack, Detroit, read the following paper, entitled

### PROPER DENTAL LEGISLATION.

Of all the subjects in the programme of this annual meeting there is none, in my opinion, which is of more importance than the one to which this day has been allotted.

In point of fact and condition, which at this time confront us, there can be none more important, standing, as we do, between two periods, when the fate of dentistry is to be decided for a long time either for the better or the worse.

This meeting, coming as it does, in the year between the biennial meetings of our State Legislature, should be so united in the upholding of the dignity of the profession and the justice of proper dental legislation, that it should put itself on record in that manner; for this year, if I read the signs of the times correctly, is the pivotal one. We must unite and act or suffer defeat, which would not only allow a retrogression in our standard, but would retard the growth of dental science, and, furthermore, would retard even proper medical legislation in this State for many years to come.

All members of the society are fully acquainted, I hope, with the strenuous efforts which were made in order to defeat the passage of an amendment to our dental law during the session of the last Legislature. This would have rendered the law practically null and void so far as the general admission of unqualified persons to practice in this State is concerned, but even when these efforts were successful we left undone the most important duty—a duty more important than the lobbying at Lansing—and that is, the rigid enforcement of the law as it stands. By so doing we could have gotten rid of the unqualified, and the fight in the Legislature could not have been renewed, but, as it is, we have merely defeated them in a preliminary skirmish—the battle is yet to come; we have merely used an antiseptic and not a germicide. The bacillus is still present and when the culture medium is once more made favorable, which will be at the next session of the Legislature, the bacillus will again be found to be active. The worst part of this disease, too, is that having it once does not cause immunity from a second attack. So, I think, it behooves us, as a society, to take some decided action on this, our dental law, and either enforce this one, making a clean sweep of the unqualified, and get rid of the trouble which will arise in the future, or if we do not this, we ought to concede the present law a failure and devote our efforts to the formulation of a law that would not be a failure. That efforts have been made to rigidly enforce the law you all know, and you all know how unsuccessful those efforts have been. Judging from this, the law, I think, must be conceded to be a failure. I do this, possibly, because of the failure of great efforts of the best men in the State to cause a conviction under the law, and the law which can not be put in force with the efforts mentioned must be a practical failure.

And now, I would like to offer some ideas and theories—perhaps they are chimerical—that they are open to criticism I have no doubt, nevertheless, I believe them to be worthy of consideration, else I should not dare to take up the time of this society.

I would like to point out what I take to be the weak points in our present law and then offer a substitute which, I think, would obviate the difficulties that we meet in the enforcement of the present one.

First: Our present law as it is in action creates two different standards—practically if not literally—the standard of the University of Michigan, and the standard of the Board of Examiners. In order to see that this statement is true, a glance at the applicants for examination, and those who have passed the examination will suffice. The majority of those who have applied and have gotten certificates from the Board of Examiners have come from colleges whose courses have been of six months' duration on the average, whereas, the University of Michigan requires nine months' course. Considering or conceding the fact that the instruction received in all colleges is the same, and to say the least, we can not give to our University of Michigan professors a lesser ability than those of other colleges, it follows, if the standard of a six months' college is sufficient to pass the Board of Examiners, that the extra three months in the University of Michigan is not a necessity ; in other words, we have conceded that the University of Michigan has a standard above that set by the Board of Examiners.

Second : The standard of the Board of Examiners varies as the personnel of the board varies. This being a fact it must follow that this law lacks dignity in court ; that in charges before the jury where the law is to be enforced, the charge that the Board of Examiners could show partiality can be used with prejudicial effect on an ordinary jury.

Third: That this Board of Examiners may, at some time, be constituted of men not qnalified for the place because it can be obtained through political influence, and when I say this I do not want any member of the present board or any member of past boards to think of personalities in this regard. Such is farthest from my thought and I merely state what I think might be a weakness.

Fourth : The provision concerning the practice of dentistry by physicians practically annuls the law in itself, as some of us saw in Detroit this spring. "A chain is no stronger than its weakest link." Anybody can register as a physician or a medical student, and as such is allowed to practice the healing art. There is no jury, even if it were composed of members of this Society, who would say that a man who is stopping and preventing pain was not doing a noble thing. And this can be said of any one who hold himself as a physician, whether he practices as a dentist or not.

Fifth : The law makes no provision for the enforcement of the law, leaving it entirely in the hands of the citizens of the State ; here it is practically pigeon-holed. Evidence and proceedings must be obtained and started entirely by some dentist in the locality in which the evil exists, and to say nothing of the time and worry and undue publicity which it gives a man it also forms ground for another charge to the jury of malicious persecution : "The silk-stockinged gentlemen who have palatial, gilded offices, and who belong to this dental union called a Dental Society, are persecuting this poor man who is honestly trying to earn a few dollars for his family ; the silk-stockings do not like competition because the poor man does his work cheaper." This is the plea to the jury which the defendant's attorney might and does use, and must we not own that it is effective?

Sixth: The present law is an injustice to an applicant who moves into the State just after a Board-examination. He has six months of uncertainty, whose fruits of labor may be scattered to the winds by failure to pass the examination. It is hardly just either to the applicant or the patients who may have come into his hands. Now, after looking at what I take to be the fallible points in the present law, I would like (as I said before) to make some suggestions along the lines of which a dental law in this State could be formed, which I humbly believe would be logical and practical. I submit the following suggestions and reasons for the same:

As a first requisite to practice in the State of Michigan, I would favor the law which would compel every man entering the profession to take an examination. This would include everybody, not even accepting graduates from our own State institution.

And the next point would be the doing away with the Board of Examiners as now constituted and placing the duties of the office in the hands of the Faculty of the University of Michigan, as officers of the State.

And, thirdly, the law ought to provide that a State officer be appointed by the Governor, whose duty it would be to act as prosecutor and complainant in these cases of violation. This officer might also be the officer whose duties would be the prosecution of cases under the Medical and Pharmacy Acts, providing likewise, that we will have a Medical Act from the next Legislature, and the probability is that we will.

Fourthly: Provisions concerning the practice of dentistry by physicians or men holding medical diplomas should not be in the law.

Fifth: This law should be made to apply to all men practicing dentistry, even those who are employed in the offices of dentists already registered should be made to pass an examination.

Having stated the provisions which I think an ideal dental law should have, I will give my reasons therefor, and as briefly as possible. In making every one take the examination, the first objection to the present law would be overcome. We could have but one standard, and that would do away with the charge of favoring any one institution, and that would be the standard of our own State institution, which, from the very nature of its connection with the State, can not be open to the charge of partiality. There are no private interests to be enhanced by holding out inducements to obtain more students.

Second: The next objection, that is the variation which is likely to be found in the standards of different Boards, would be met, as there would be no standard so unvarying as the University.

Third: The personnel of the Board would necessarily be of the highest, and subject to none of the changes which could take place in the present condition of affairs.

Fourth: The point concerning the legality of a medical diploma allowing dental practice would be virtually overcome for the State itself, through its institution and appropriations for the same, takes for granted that a dental education is an education of a special kind, else it would not appropriate moneys for a dental school when it already was supporting a medical school. Thus this point would be annulled, and furthermore, if everybody were compelled to take an examination, it would follow that a medical diploma would carry no weight. It would also eliminate the question as to whether dentistry is a specialty of medicine or not, and would allow dentistry, like all self-respecting tubs, to stand upon its own bottom.

Another point to be considered is, that the Faculty of the University would always be in session and applicants could or should be examined at any time, thus preventing the lapse of six months' time, which, as I pointed out before, might be unjust to both applicants and patients.

The provision for a public prosecuting-officer of the State is, I think, of sufficient weight to commend itself—that the University should be the standard is, in my opinion, the best thing that could be for the profession in this State and elsewhere. It adds dignity to the work and study of dentistry. It is kept by State funds; it employs only the best men as teachers who devote almost the whole of their time to it; that they would be impartial in their examinations could not, I think, be fairly contradicted. From what motives could or would they be otherwise Their interests are in no wise at stake. The school which they help by their teaching always has had a sufficient number of students. Their private practice in dentistry is not of such great proportions that the coming in of new practitoners to their neighborhood would influence them.

Perhaps it may be said that such a law would be harsh upon men who have been some time out of college, and who had been in practice several years before making application, but here it is supposed that the Faculty would be broad enough to take into consideration this fact and weigh their qualifications justly, even so it would be similar to the stand taken by the present Board. To any considering this, a glance over the list of applicants for the last few years will show that the exception is the man who makes application after having been in practice for over five years in some other State.

The papers by Drs. Corbin and Diack were very fully discussed by Drs. Metcalf, Daick, Fields, Root, Lathrop, Loeffler, Corbin and Taft, and at the conclusion of the discussion the Secretary moved the adoption of the following resolution:

*Resolved*, That the Committee appointed on Resolutions, which is a permanent committee, be requested to consider the matter of appointing a Public Prosecutor, if I may so call him, and make a report at the morning session or some time to-morrow.

Carried.

On motion, the meeting adjourned till morning.

